# Biokinetics of subacutely co-inhaled same size gold and silver nanoparticles

**DOI:** 10.1186/s12989-023-00515-z

**Published:** 2023-03-31

**Authors:** Philku Lee, Jin Kwon Kim, Mi Seong Jo, Hoi Pin Kim, Kangho Ahn, Jung Duck Park, Mary Gulumian, Günter Oberdörster, Il Je Yu

**Affiliations:** 1 Institute for Genomics, Biocomputing and Biotechnology, Starkville, MS USA; 2 HCT CO., LTD, Seoicheon-Ro 578 Beon-Gil, Majang-Myeon, Icheon, 17383 Korea; 3 Hanyang University, Ansan, Korea; 4 College of Medicine, Chung-Ang University, Seoul, Korea; 5 Haematology and Molecular Medicine, University of the Witwatersrand, Johannesburg, South Africa; 6 Water Research Group, Unit for Environmental Sciences and Management, North-West University, Potchefstroom, South Africa; 7 Department of Environmental Medicine, University of Rochester, Rochester, NY USA

**Keywords:** Biokinetics, Toxicokinetics, Translocation, Silver nanoparticles, Gold nanoparticles, Elimination, Tissue distribution, Clearance, Co-inhalation exposure

## Abstract

**Background:**

Toxicokinetics of nanomaterials, including studies on the absorption, distribution, metabolism, and elimination of nanomaterials, are essential in assessing their potential health effects. The fate of nanomaterials after inhalation exposure to multiple nanomaterials is not clearly understood.

**Methods:**

Male Sprague–Dawley rats were exposed to similar sizes of silver nanoparticles (AgNPs, 10.86 nm) and gold nanoparticles (AuNPs, 10.82 nm) for 28 days (6-h/day, 5-days/week for four weeks) either with separate NP inhalation exposures or with combined co-exposure in a nose-only inhalation system. Mass concentrations sampled from the breathing zone were AuNP 19.34 ± 2.55 μg/m^3^ and AgNP 17.38 ± 1.88 μg/m^3^ for separate exposure and AuNP 8.20 μg/m^3^ and AgNP 8.99 μg/m^3^ for co-exposure. Lung retention and clearance were previously determined on day 1 (6-h) of exposure (E-1) and on post-exposure days 1, 7, and 28 (PEO-1, PEO-7, and PEO-28, respectively). In addition, the fate of nanoparticles, including translocation and elimination from the lung to the major organs, were determined during the post-exposure observation period.

**Results:**

AuNP was translocated to the extrapulmonary organs, including the liver, kidney, spleen, testis, epididymis, olfactory bulb, hilar and brachial lymph nodes, and brain after subacute inhalation and showed biopersistence regardless of AuNP single exposure or AuNP + AgNP co-exposure, showing similar elimination half-time. In contrast, Ag was translocated to the tissues and rapidly eliminated from the tissues regardless of AuNP co-exposure. Ag was continually accumulated in the olfactory bulb and brain and persistent until PEO-28.

**Conclusion:**

Our co-exposure study of AuNP and AgNP indicated that soluble AgNP and insoluble AuNP translocated differently, showing soluble AgNP could be dissolved into Ag ion to translocate to the extrapulmonary organs and rapidly removed from most organs except the brain and olfactory bulb. Insoluble AuNPs were continually translocated to the extrapulmonary organs, and they were not eliminated rapidly.

**Supplementary Information:**

The online version contains supplementary material available at 10.1186/s12989-023-00515-z.

## Background

Nanoparticles have been known to be translocated to other organs after exposure and eliminated from the organs. Major exposure pathways are through inhalation, ingestion, or injection. The lung-deposited aerosol state nanoparticles are readily translocated to extrapulmonary organs and other target organs by different routes and mechanisms. One is systemic blood circulation or via lymphatic ducts after crossing the air–liquid barrier to the respiratory epithelial layer and interstitial tissues. Another is by sensory nerve endings in the airway epithelia, followed by axonal translocation to ganglionic and CNS structures [1]. Previously, other groups have demonstrated two different pathways of clearance from the lung that exist after subacute co-exposing soluble silver nanoparticles (AgNP) and insoluble gold nanoparticle (AuNP) and thereafter post-exposure observation (PEO) period. Insoluble AuNP is mechanically cleared, while soluble AgNP is cleared initially chemically by dissolution and by mechanically after the dissolved Ag ions form insoluble secondary AgNP with biogenic molecules [2–5]. The clearance mechanisms for inhaled solid particles in the respiratory tract are physical clearance processes (translocation), including mucociliary movement, macrophage phagocytosis, epithelial endocytosis, lymphatic drainage, blood circulation, and sensory neurons, and chemical clearance processes including dissolution, leaching, and protein binding [1].

Translocation or tissue distribution of AgNP after inhalation exposure has been studied, exhibiting wide tissue distribution [6–10]. Several lung retention studies after AgNP inhalation suggest that AgNP which is soluble can be existed as AgNP, Ag ion and secondary insoluble AgNP in the lung [2–4]. Extrapulmonary translocation or tissue distribution of AuNP after inhalation also showed wide tissue distribution to a less degree than AgNP [3, 11–14]. Workers in the workplace and consumers using nanomaterial-containing products are not likely to be exposed to one kind of nanomaterials; rather, they could be co-exposed to multiple nanoparticles, including soluble and insoluble nanomaterials. Lung retention and clearance of co-inhalation exposure of soluble nanoparticles AgNP and insoluble nanoparticles AuNP have been studied in a previous study by Kim et al. [3]. The study indicated that the clearance of AgNPs follows a two-compartment model of fast and slow dissolution rates, while the clearance of AuNPs could be described by a one-compartment model with a longer half-time. The co-exposure of AuNPs + AgNPs showed that the clearance of AgNPs was altered by the presence of AuNPs, perhaps due to some interaction between AgNP and AuNP affecting dissolution and/or mechanical clearance of AgNP in vivo [3].

Extrapulmonary translocation of AgNP and AuNP after co-inhalation exposure of AgNP and AuNP has not been studied; furthermore, the elimination of AgNP and AuNP from the organs after co-inhalation exposure also has not been studied. Understanding the translocation and clearance of insoluble nanoparticles such as AuNP and soluble nanoparticles such as AgNP after co-inhalation exposure will enhance our knowledge of the toxicokinetics of nanomaterials. Thus, the aim of this paper is to show that insoluble AuNPs and soluble AgNPs after inhalation alone or in combination is their translocation to extrapulmonary organs and thereafter their elimination. Also, the effect of one particle type on the other on these processes upon co-exposure. In this report, we have investigated the fate of translocated AgNP and AuNP after co-exposure.

## Results

### Characterization of AgNP and AuNP aerosols in inhalation chambers

The total number concentrations, count median diameter (CMD), geometric standard deviation (GSD), and surface area of the AgNPs, AuNPs, and AuNPs + AgNPs measured by the DMAS during the exposure period are published and presented in Additional file 1: Table S1 [3]. FE-TEM revealed non-agglomerated particles, and TEM-EDS identified AgNP and AuNP particles in each chamber (Additional file 1: Figure S1) [3]. The mass concentrations analyzed by AAS via filter sampling were 17.38 ± 1.88 μg/m^3^ for AgNPs, 19.34 ± 2.55 μg/m^3^ for AuNPs for single exposure, and 8.99 ± 1.77 AgNPs + 8.20 ± 1.05 AuNPs for AuNP + AgNP for co-exposure, while the mass concentrations estimated by DMAS were 10.12 ± 0.71 μg/m^3^ for AgNPs and 17.68 ± 1.1.69 μg/m^3^ for AgNPs, respectively. TEM indicated that the AgNPs, AuNPs, and AuNPs + AgNPs were the particle diameters log-normally distributed between 6 and 30 nm. The CMD and GSD measurements were 10.40 nm and 1.36, respectively, for the AuNPs, 9.48 nm and 1.49, respectively, for the AgNPs, and 9.00 nm and 1.19, respectively, for the AuNP + AgNP coexposure (Additional file 1: Table S1, Fig. S2).

### Organ retention after AuNP exposure and AuNP + AgNP co-exposure

Our previous study [2] investigated the lung burden of rats that were exposed to biosoluble silver nanoparticles (AgNPs, 10.86 nm) and to biopersistent gold nanoparticles (AuNPs, 10.82 nm) for 28 days (6-h/day, 5-days/week for 4 weeks) either with separate NP inhalation exposures or with combined co-exposure. After 28-day of AuNP or AuNP + AgNP coexposure, a 97.9 and 97.1% of Au were retained, respectively (Additional file 1: Table S2). For Single AuNP exposure, an elimination half-time (T_1/2_) was 81.5 days, while coexposure with AgNP reduced the AuNP T_1/2_ to 54.2 days (Table 1).

**Table 1:** Elimination half-times (T_1/2_) of Au and Ag in organs (days)

Exposure	AuNP (days)	AuNP + AgNP (days)	AgNP (days)	AuNP + AgNP (days)
Measured	Au		Ag	
Lung*	81.5	54.2	3.1 (fast) 48.5 (slow)	2.2 (fast) 28.4 (slow)
Liver	192.60	221.4	1.4	2.5
Kidneys	Accumulation	Accumulation	Accumulation	Accumulation
Spleen	177.0	Accumulation	Accumulation	NE
Testes	NE	Accumulation	Accumulation	Accumulation
Epididymis	Accumulation	Accumulation	29.9 days	Accumulation
Olfactory bulb	20.4	NE	75.1	17.8
Eyes	25.8	NE	68.7	128.6
Brain	NE	NE	Accumulation	108.9
Hilar lymph nodes	65.3	25.2	11.4	2.8
Brachial lymph nodes	10.8	11.1	59.7	53.5
Thymus	17.7	NE	89.5	Accumulation
Blood	2.2	NE	NE	NE

NE, not eliminated; Accumulated, Au or Ag concentration is increased during PEO

*Kim et al. [3]

**Fig. 1:**
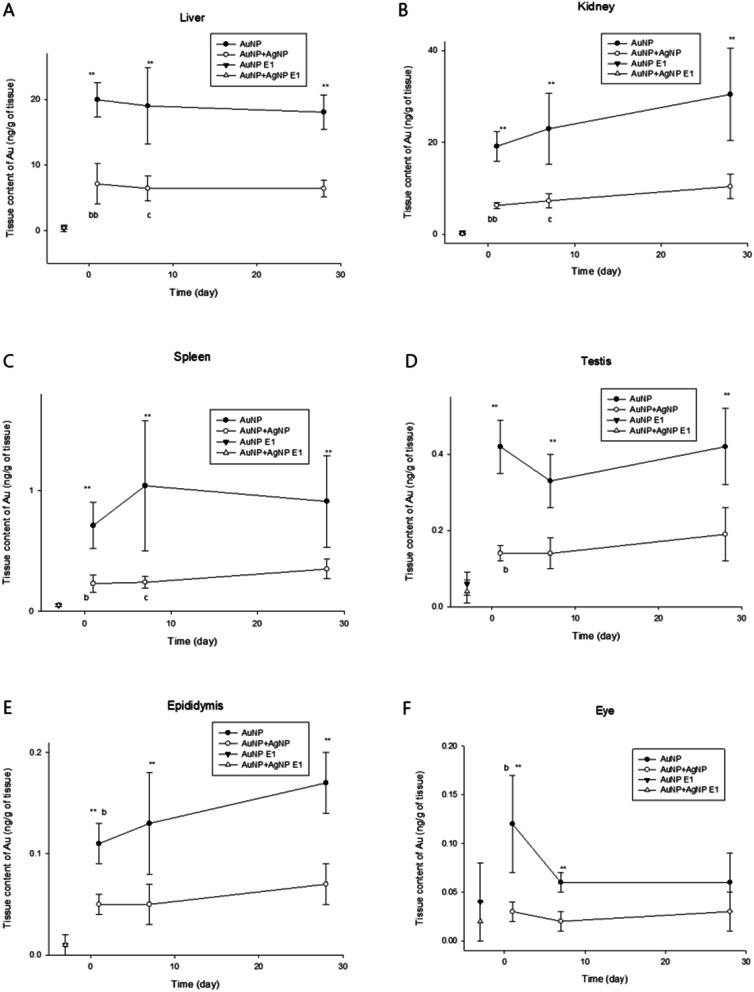

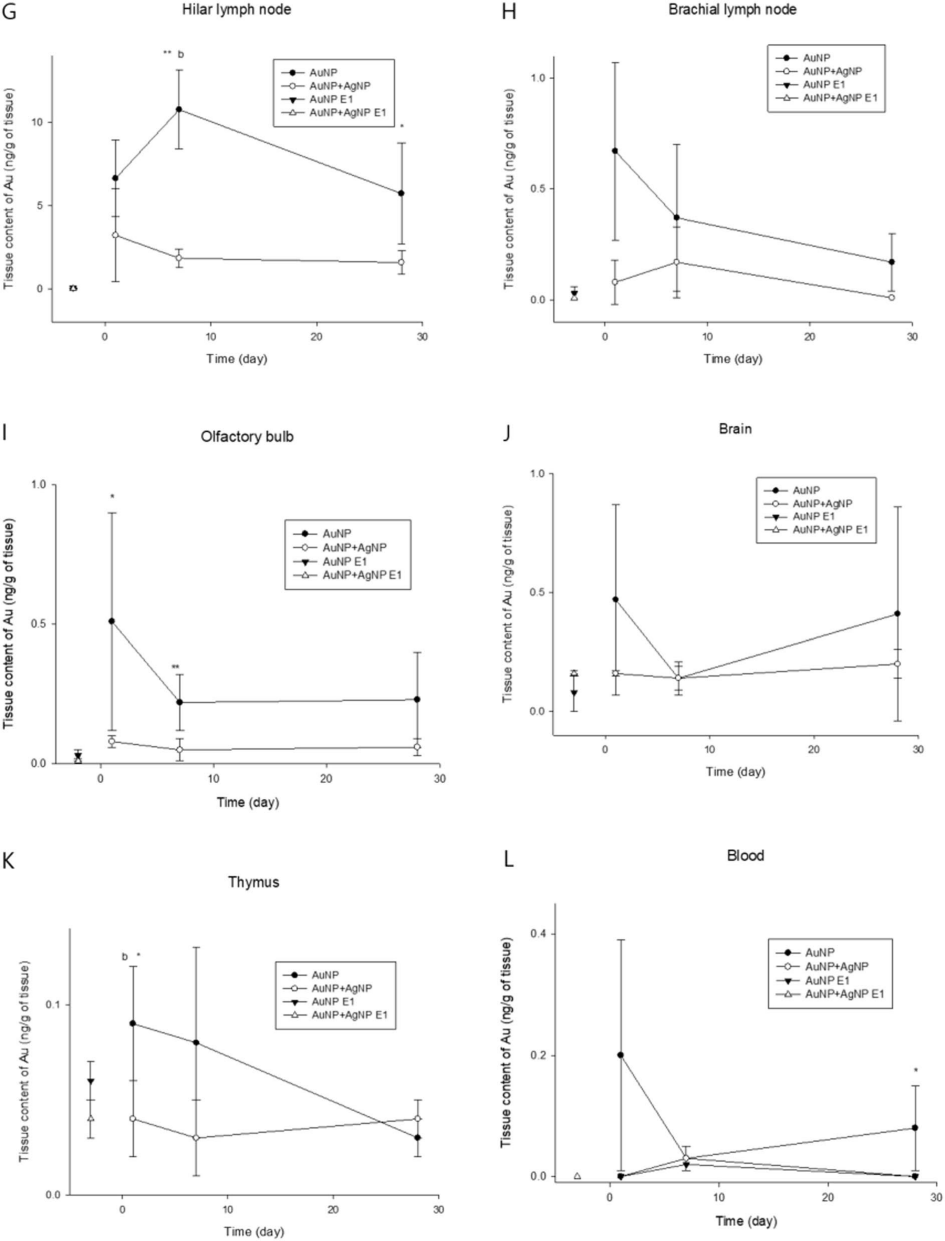
Tissue Au concentration after 28-day inhalation exposure and post exposure period. E1, exposure 1 day. ^b^*P* < 0.05, compared with PEO-1 and PEO-28; ^c^*P* < 0.05, compared with PEO-7 and PEO-28; ^bb^*P* < 0.01, compared with PEO-1 and PEO-28; Where (t-test) *P < 0.05, and **P < 0.01

Organ concentrations of Au and Ag after subacute exposure and post-exposure observation (PEOs) periods were presented in Table 2 and 3, respectively. AuNP was continually accumulated in the lung with very low elimination during the subacute exposure period of either AuNP alone or AuNP + AgNP coexposure. Liver, kidney, and hilar lymph nodes showed a considerable amount of extrapulmonary translocation of AuNP (Table 2). Other organs, including the spleen, testis epididymis, thymus, olfactory bulb, brachial lymph node, brain, eye, and blood, showed very low translocation with sub-nanogram ranges (Fig. 1). Comparing the hilar lymph node to the brachial lymph node, the lymphatic translocation of AuNP showed increased translocation of AuNP proximal to the lung compared with distal to the lung. As seen in Fig. 1, AuNPs exposed either AuNP alone (AuNP E-1, closed triangle) or together with AgNP (AuNP + AgNP E1, open triangle) were continually accumulated in the extrapulmonary organs from the exposure 1-day (E-1) to post-exposure 1-day (PEO-1). AuNPs were either eliminated slowly from the tissues or accumulated in the most examined organs, including the liver (T_1/2_ 192.6 days for AuNP exposure; 221.4 days coexposure), kidney, Spleen (T_1/2_ 177 days), testis, epididymis, and brain, either after 28-days of AuNP single exposure (closed circle) or AuNP + AgNP coexposure (open circle) (Table 1, Fig. 1, Table 2). The kidney, spleen testis, and epididymis showed accumulation of Au with AuNP alone and AuNP + AgNP coexposure. The eyes, brachial lymph node, hilar lymph node, and olfactory bulb showed a persistent tissue concentration of Au after AuNP or AuNP + AgNP coexposure. The accumulated AuNPs were not easily cleared from most organs, including the liver, kidney, spleen testis, epididymis, hilar lymph nodes, and brain (Table 1, Fig. 1, Table 2). The kidney, spleen, testis, and epididymis showed accumulation of Au alone or with AgNP. The olfactory bulb, eyes, brachial lymph nodes, and thymus showed some level of clearance with AuNP exposure but not with AgNP coexposure, even after PEO-28. These organs showing somewhat faster elimination compared with slower elimination organs could be due to the small number of samples, the lower concentration (< ng), and the resulting deviation. When the AuNP elimination was plotted with organ elimination kinetics, Au elimination in the liver, kidney, spleen, epididymis, olfactory bulb, eye, and brachial lymph node showed similar elimination regardless of AuNP exposure alone or AuNP + AgNP coexposure (Table 2, Additional file 1: Fig. S3). Other organs, such as the hilar lymph node, testis, thymus, and brain, showed different retention between AuNP alone and AuNP + AgNP coexposure. Interestingly, the brain exposed to AuNP alone showed V-shaped elimination kinetics showing reduced retention at PEO-7, but the retention increased again at PEO-28 (Additional file 1: Fig. S3). The hilar lymph node, the first organ of translocation from the lung except blood, showed a different pattern showing increased retention of PEO-7 and PEO-28 when comparing AuNP alone with AuNP + AgNP coexposure, indicating continuous translocation of AuNP from the lung tissue to the lymph node even after termination of inhalation exposure (Fig. 1).

**Table 2:** The organ distribution for gold after inhaled of AuNP at exposure 1 day (E−1), post-exposure observation 1 day (PEO-1), PEO-7 days and PEO-28 days (mean ± S.E; 4 animals of exposure 1 day; 5 animals of PEO-1, PEO-7 and PEO-28)

AuNP	Liver			Kidney			Spleen			Testis			Epididymis		
	ng/g^A^	Organ wt (g)^B^	Au/organ (ng)^C^	ng/g^A^	Organ wt (g)^B^	Au/organ (ng)^C^	ng/g^A^	Organ wt (g)^B^	Au/organ (ng)^C^	ng/g^A^	Organ wt (g)^B^	Au/organ (ng)^C^	ng/g^A^	Organ wt (g)^B^	Au/organ (ng)^C^
E−1	0.002 ± -	8.99 ± 0.137	0.017 ± -	0.231 ± 0.028	1.238 ± 0.033	0.286 ± 0.035	N/D	0.641 ± 0.086	N/D	0.032 ± 0.004	1.463 ± 0.062	0.048 ± 0.007	0.028 ± 0.008	0.280 ± 0.032	0.008 ± 0.004
PEO1	1.913 ± 0.125	10.471 ± 0.368	19.956 ± 1.170	14.312 ± 0.978	1.337 ± 0.025	19.158 ± 1.444	1.061 ± 0.113	0.676 ± 0.040	0.712 ± 0.113	0.261 ± 0.018	1.614 ± 0.041	0.422 ± 0.033	0.200 ± 0.018	0.539 ± 0.061	0.108 ± 0.011^b^
PEO7	1.840 ± 0.250	10.408 ± 0.962	19.007 ± 2.621	15.331 ± 1.302	1.475 ± 0.144	22.965 ± 3.432	1.416 ± 0.308	0.723 ± 0.040	1.042 ± 0.308	0.212 ± 0.012	1.528 ± 0.085	0.327 ± 0.032	0.246 ± 0.036	0.515 ± 0.054	0.129 ± 0.023
PEO 28	1.473 ± 0.113	12.332 ± 0.426	18.062 ± 1.182	18.193 ± 2.918	1.712 ± 0.096	30.461 ± 4.466	0.920 ± 0.133	0.972 ± 0.068	0.907 ± 0.133	0.248 ± 0.032	1.741 ± 0.080	0.423 ± 0.045	0.264 ± 0.020	0.658 ± 0.024	0.173 ± 0.012

Co-exposure	Liver			Kidney			Spleen			Testis			Epididymis		
E−1	0.034 ± 0.007	9.362 ± 1.021	0.341 ± 0.097	0.144 ± 0.040	1.226 ± 0.110	0.166 ± 0.044	N/D	0.589 ± 0.074	0.166 ± 0.044	0.032 ± 0.004	1.528 ± 0.044	0.049 ± 0.006	0.048 ± 0.011	0.296 ± 0.012	0.014 ± 0.006
PEO1	0.787 ± 0.174	9.237 ± 0.529	7.103 ± 1.371	4.990 ± 0.154	1.257 ± 0.037	6.278 ± 0.297^bb^	0.406 ± 0.065	0.571 ± 0.023	0.230 ± 0.065^b^	0.086 ± 0.005	1.638 ± 0.089	0.140 ± 0.009^b^	0.094 ± 0.008	0.482 ± 0.058	0.045 ± 0.005
PEO7	0.700 ± 0.157	9.987 ± 0.919	6.433 ± 0.843	5.731 ± 0.723	1.300 ± 0.074	7.298 ± 0.694^c^	0.363 ± 0.029	0.656 ± 0.043	0.238 ± 0.029^c^	0.092 ± 0.015	1.611 ± 0.087	0.145 ± 0.017	0.091 ± 0.023	0.550 ± 0.051	0.048 ± 0.011
PEO 28	0.471 ± 0.045	13.905 ± 0.894	6.490 ± 0.565	6.534 ± 0.847	1.609 ± 0.053	10.404 ± 1.186	0.359 ± 0.058	1.054 ± 0.142	0.355 ± 0.058	0.115 ± 0.011	1.677 ± 0.021	0.193 ± 0.016	0.101 ± 0.010	0.675 ± 0.080	0.069 ± 0.008

AuNP	Thymus			Olfactory bulb			Hilar lymph node			Brachial lymph node			Brain		
E−1	N/D	0.728 ± 0.060	N/D	0.314 ± 0.091	0.082 ± 0.004	0.026 ± 0.008	5.018 ± 1.434	0.019 ± 0.002	0.071 ± 0.021	0.416 ± 0.218	0.082 ± 0.004	0.032 ± 0.016	0.042 ± 0.019	2.053 ± 0.039	0.083 ± 0.038
PEO1	0.236 ± 0.053	0.418 ± 0.075	0.091 ± 0.015^b^	5.899 ± 2.572	0.097 ± 0.013	0.512 ± 0.176	578.442 ± 153.265	0.013 ± 0.002	6.633 ± 1.147	6.967 ± 1.480	0.097 ± 0.013	0.666 ± 0.179	0.225 ± 0.085	2.113 ± 0.047	0.472 ± 0.181
PEO7	0.186 ± 0.067	0.408 ± 0.084	0.074 ± 0.025	3.999 ± 0.900	0.059 ± 0.013	0.219 ± 0.044	705.582 ± 375.512	0.029 ± 0.009	10.761 ± 1.180^c^	5.637 ± 1.464	0.059 ± 0.013	0.372 ± 0.147	0.055 ± 0.026	2.197 ± 0.037	0.118 ± 0.055
PEO 28	0.052 ± 0.004	0.397 ± 0.022	0.023 ± 0.003	3.500 ± 0.480	0.059 ± 0.014	0.227 ± 0.075	156.841 ± 30.359	0.035 ± 0.004	5.724 ± 1.518	2.908 ± 0.754	0.059 ± 0.014	0.174 ± 0.060	0.198 ± 0.105	2.312 ± 0.041	0.465 ± 0.249

Co-exposure	Thymus			Olfactory bulb			Hilar lymph node			Brachial lymph node			Brain		
E−1	N/D	0.573 ± 0.043	N/D	0.174 ± 0.135	0.061 ± 0.008	0.008 ± 0.006	2.042 ± 0.342	0.019 ± 0.005	0.018 ± 0.005	N/D	0.061 ± 0.008	N/D	N/D	2.093 ± 0.015	N/D
PEO1	0.142 ± 0.074	0.430 ± 0.055	0.043 ± 0.016	0.812 ± 0.135	0.103 ± 0.008	0.081 ± 0.011	211.322 ± 128.818	0.022 ± 0.005	3.216 ± 1.390	0.932 ± 0.448	0.103 ± 0.008	0.101 ± 0.051	0.086 ± -	2.079 ± 0.023	0.180 ± -
PEO7	0.013 ± -	0.498 ± 0.044	0.005 ± -	0.490 ± 0.179	0.108 ± 0.008	0.056 ± 0.023	57.529 ± 11.634	0.034 ± 0.004	1.840 ± 0.270	1.998 ± 0.686	0.108 ± 0.008	0.206 ± 0.077	0.029 ± -	2.118 ± 0.062	0.060 ± -
PEO 28	N/D	0.466 ± 0.044	N/D	0.511 ± 0.089	0.111 ± 0.008	0.058 ± 0.012	31.982 ± 6.204	0.050 ± 0.005	1.585 ± 0.362	N/D	0.111 ± 0.008	N/D	0.136 ± -	2.277 ± 0.032	0.309 ± -

	Eye			Blood		
*AuNP*						
E−1	0.096 ± 0.063	0.365 ± 0.033	0.041 ± 0.030	0.020 ± 0.007	0.5 mL	0.010 ± 0.004
PEO1	0.276 ± 0.042	0.421 ± 0.023	0.119 ± 0.024^b^	0.392 ± 0.171	0.5 mL	0.196 ± 0.095
PEO7	0.167 ± 0.019	0.382 ± 0.013	0.063 ± 0.006	0.062 ± 0.019	0.5 mL	0.031 ± 0.011
PEO 28	0.121 ± 0.028	0.478 ± 0.031	0.057 ± 0.012	0.168 ± 0.065	0.5 mL	0.084 ± 0.036
*Co-exposure*						
E−1	0.069 ± 0.064	0.334 ± 0.020	0.021 ± 0.020	0.012 ± 0.005	0.5 mL	0.006 ± 0.002
PEO1	0.079 ± 0.014	0.376 ± 0.016	0.029 ± 0.005	0.001 ± 0.000	0.5 mL	0.001 ± 0.000
PEO7	0.065 ± 0.016	0.370 ± 0.006	0.024 ± 0.006	0.053 ± 0.017	0.5 mL	0.026 ± 0.009
PEO 28	0.047 ± 0.011	0.561 ± 0.090	0.026 ± 0.007	0.005 ± 0.003	0.5 mL	0.002 ± 0.001

A statistics analysis was used Anova and test.

A, samples were analyzed using by ICP-MS and calculated following ppb (ug/L) × final volume (5 mL) × dilution factor (10 times) / organ weight.

B, Organ weights were measured using by micro balance. Before measure weight, washed blood to di-water and wiped up to paper.

C, The gold concentration (ng/g) × organ weight (g); N/D was analyzed sample silver concentration lower than compare with control.

Statistics were analyzed Anova (multiple comparison analysis) and *t*-test. This result was described in Table that Anova was from a to cc and *t*-test was * and **.

^a^*P* < 0.05, compared with PEO-1 and PEO-7

^b^*P* < 0.05, compared with PEO-1 and PEO-28

^aa^*P* < 0.01, compared with PEO-1 and PEO-7’

^bb^*P* < 0.01, compared with PEO-1 and PEO-28

*P < 0.05, compared with PEO-1 and PEO-7

**P < 0.01, compared with PEO-1 and PEO-28

**Table 3:** The organ distribution for silver after inhaled of AgNP at exposure 1 day (E-1), post-exposure observation 1 day (PEO-1), PEO-7 days and PEO-28 days (mean ± S.E; 4 animals of exposure 1 day; 5 animals of PEO-1, PEO-7 and PEO-28). A statistics analysis was used Anova and test

AgNP	Liver			Kidney			Spleen			Testis			Epididymis		
	ng/g^A^	Organ wt (g)^B^	Au/organ (ng)^C^	ng/g^A^	Organ wt (g)^B^	Au/organ (ng)^C^	ng/g^A^	Organ wt (g)^B^	Au/organ (ng)^C^	ng/g^A^	Organ wt (g)^B^	Au/organ (ng)^C^	Au/organ (ng)^C^	ng/g^A^	Organ wt (g)^B^
E-1	7.981 ± 1.458	8.767 ± 0.332	69.407 ± 11.214	0.767 ± 0.223	1.175 ± 0.061	0.926 ± 0.327	N/D	0.657 ± 0.131	N/D	0.261 ± 0.130	1.488 ± 0.174	0.344 ± 0.142	N/D	0.339 ± 0.022	N/D
PEO1	7.235 ± 1.907	9.418 ± 0.467	66.974 ± 18.033*	0.486 ± 0.266	1.192 ± 0.043	0.623 ± 0.376	0.724 ± 0.493	0.600 ± 0.013	0.426 ± 0.118	0.284 ± 0.083	1.511 ± 0.086	0.431 ± 0.126	0.166 ± 0.030	0.506 ± 0.035	0.078 ± 0.014^a^
PEO7	0.215 ± 0.149	10.717 ± 0.430	2.573 ± 1.593	0.444 ± 0.444	1.330 ± 0.053	0.575 ± 0.575	1.279 ± -	0.723 ± 0.040	0.836 ± -	0.195 ± 0.082	1.550 ± 0.108	0.257 ± 0.112	0.641 ± 0.218	0.624 ± 0.065	0.359 ± 0.112^ aa^
PEO 28	N/D	11.768 ± 0.180	N/D	N/D	1.449 ± 0.056	N/D	N/D	0.972 ± 0.068	N/D	0.168 ± 0.168	1.677 ± 0.046	0.272 ± 0.272	0.102 ± 0.010	0.610 ± 0.047	0.062 ± 0.008

Co-exposure	Liver			Kidney			Spleen			Testis			Epididymis		
E-1	2.238 ± 0.628	9.362 ± 1.021	22.598 ± 8.612	0.198 ± -	1.226 ± 0.110	0.244 ± -	N/D	0.589 ± 0.074	0.166 ± 0.044	N/D	1.528 ± 0.044	N/D	N/D	0.296 ± 0.012	N/D
PEO1	4.912 ± 1.988	9.237 ± 0.529	42.262 ± 14.830	0.372 ± 0.155	1.257 ± 0.037	0.469 ± 0.190	1.832 ± 0.197	0.571 ± 0.023	0.999 ± 0.099	0.199 ± 0.050	1.638 ± 0.089	0.313 ± 0.069	0.008 ± 0.008	0.482 ± 0.058	0.004 ± 0.004
PEO7	0.894 ± 0.673	9.987 ± 0.919	7.098 ± 4.499	0.375 ± -	1.300 ± 0.074	0.537 ± -	0.699 ± 0.111	0.656 ± 0.043	0.492 ± 0.126	0.279 ± 0.061	1.611 ± 0.087	0.476 ± 0.125	0.235 ± 0.077	0.550 ± 0.051	0.125 ± 0.046
PEO 28	N/D	13.905 ± 0.894	N/D	N/D	1.609 ± 0.053	N/D	N/D	1.054 ± 0.142	N/D	0.132 ± -	1.677 ± 0.021	0.211 ± -	0.327 ± 0.070	0.675 ± 0.080	0.213 ± 0.076

AgNP	Thymus			Olfactory bulb			Hilar lymph node			Brachial lymph node			Brain		
	ng/g^A^	Organ wt (g)^B^	Au/organ (ng)^C^	ng/g^A^	Organ wt (g)^B^	Au/organ (ng)^C^	ng/g^A^	Organ wt (g)^B^	Au/organ (ng)^C^	ng/g^A^	Organ wt (g)^B^	Au/organ (ng)^C^	Au/organ (ng)^C^	ng/g^A^	Organ wt (g)^B^
E-1	N/D	0.597 ± 0.050	N/D	2.842 ± 1.175	0.044 ± 0.008	0.098 ± 0.030	62.109 ± 8.273	0.004 ± 0.001	0.206 ± 0.039	3.009 ± 1.858	0.044 ± 0.008	0.095 ± 0.048	N/D	2.093 ± 0.015	N/D
PEO1	N/D	0.471 ± 0.098	N/D	25.646 ± 4.401	0.059 ± 0.002	1.552 ± 0.303	16.337 ± 13.087	0.014 ± 0.003	0.196 ± 0.157	8.119 ± 4.437	0.059 ± 0.002	0.495 ± 0.279	1.219 ± 0.151	2.049 ± 0.061	2.507 ± 0.336
PEO7	0.482 ± -	0.497 ± 0.031	0.280 ± -	24.925 ± 3.328	0.063 ± 0.003	1.559 ± 0.200	4.055 ± 1.298	0.042 ± 0.006	0.146 ± 0.044	18.072 ± 9.080	0.063 ± 0.003	1.152 ± 0.504	0.941 ± 0.121	2.192 ± 0.040	2.045 ± 0.231
PEO 28	N/D	0.393 ± 0.034	N/D	19.160 ± 0.567	0.065 ± 0.004	1.245 ± 0.108	N/D	0.033 ± 0.006	N/D	7.073 ± 5.843	0.065 ± 0.004	0.387 ± 0.306	1.195 ± 0.115	2.147 ± 0.046	2.568 ± 0.262

Co-exposure	Thymus			Olfactory bulb			Hilar lymph node			Brachial lymph node			Brain		
E-1	0.146 ± -	0.573 ± 0.043	0.146 ± -	1.180 ± 0.394	0.061 ± 0.008	0.065 ± 0.026	15.323 ± 6.319	0.019 ± 0.005	0.105 ± 0.068	N/D	0.061 ± 0.008	N/D	N/D	2.093 ± 0.015	N/D
PEO1	0.958 ± 0.279	0.430 ± 0.055	0.277 ± 0.044	9.369 ± 2.395	0.103 ± 0.008	0.916 ± 0.192^b^	11.537 ± 6.950	0.022 ± 0.005	0.143 ± 0.079	0.192 ± -	0.103 ± 0.008	0.018 ± -	0.284 ± 0.105	2.079 ± 0.023	0.593 ± 0.221
PEO7	0.252 ± -	0.498 ± 0.044	0.152 ± -	4.443 ± 0.786	0.108 ± 0.008	0.484 ± 0.105	0.242 ± -	0.034 ± 0.004	0.004 ± -	0.303 ± -	0.108 ± 0.008	0.035 ± -	0.752 ± 0.280	2.118 ± 0.062	1.536 ± 0.553
PEO 28	N/D	0.466 ± 0.044	N/D	3.302 ± 0.378	0.111 ± 0.008	0.372 ± 0.061	N/D	0.050 ± 0.005	N/D	N/D	0.111 ± 0.008	N/D	0.510 ± 0.151	2.277 ± 0.032	1.144 ± 0.322

AgNP	Eye			Blood		
	ng/g^A^	Organ wt (g)^B^	Au/organ (ng)^C^	ng/g^A^	Organ wt (g)^B^	Au/organ (ng)^C^
E-1	0.989 ± 0.509	0.333 ± 0.005	0.320 ± 0.161	0.027 ± 0.004	0.5	0.013 ± 0.004
PEO1	0.218 ± -	0.355 ± 0.017	0.083 ± -	0.036 ± 0.008	0.5	0.018 ± 0.004*
PEO7	1.253 ± 0.211	0.389 ± 0.007	0.476 ± 0.091	0.004 ± 0.002	0.5	0.002 ± 0.001
PEO 28	0.077 ± -	0.419 ± 0.016	0.034 ± -	N/D	0.5	N/D

Co-exposure	Eye			Blood		
E-1	N/D	0.334 ± 0.020	N/D	0.008 ± 0.001	0.5	0.004 ± 0.001
PEO1	N/D	0.376 ± 0.016	N/D	0.016 ± 0.002	0.5	0.008 ± 0.001**
PEO7	N/D	0.370 ± 0.006	N/D	0.004 ± 0.001	0.5	0.002 ± 0.001
PEO 28	N/D	0.561 ± 0.090	N/D	0.008 ± 0.001	0.5	0.004 ± 0.001

A, samples were analyzed using by ICP-MS and calculated following ppb (ug/L) x final volume (5 mL) x dilution factor (10 times) / organ weight.

B, Organ weights were measured using by micro balance. Before measure weight, washed blood to di-water and wiped up to paper.

C, The silver concentration (ng/g) x organ weight (g)

N/D was analyzed sample silver concentration lower than compare with control.

A statistics were analyzed Anova (multiple comparison analysis of Dennett test) and t-test. This result was described in Table that Anova was from a to cc and t-test was * and **.

Where (Anova)

^a^*P* < 0.05, compared with PEO-1 and PEO-7

^b^*P* < 0.05, compared with PEO-1 and PEO-28

^aa^*P* < 0.01, compared with PEO-7 and PEO-28

Where (t-test)

*P < 0.05, and **P < 0.01

### AgNP exposure and AgNP and AuNP co-exposure

In contrast to AuNP exposure, between 34 and 49% of deposited Ag in the lung was estimated to be solubilized and removed from the lung within 5 days after 28-days of AgNP exposure and AuNP-AgNP co-exposure. Estimated insoluble AgNPs were retained at 66.1% and 51.2% after 28-days of AgNP and AgNP + AuNP co-exposure, respectively (Additional file 1: Table S2). The elimination of single AgNP exposure and AuNP + AgNP co-exposure showed two phases for Ag elimination; fast and slow. The fast-elimination T_1/2_ after single exposure was 3.1 days, and the slow-elimination T_1/2_ single exposure was 48.5 days. AuNP + AgNP co-exposure also showed 2 phases of Ag elimination in the lung; fast and slow, where the fast-elimination T_1/2_ was 2.2 days, and the slow-elimination T_1/2_ was 28.4 days (Table 1).

**Fig. 2:**
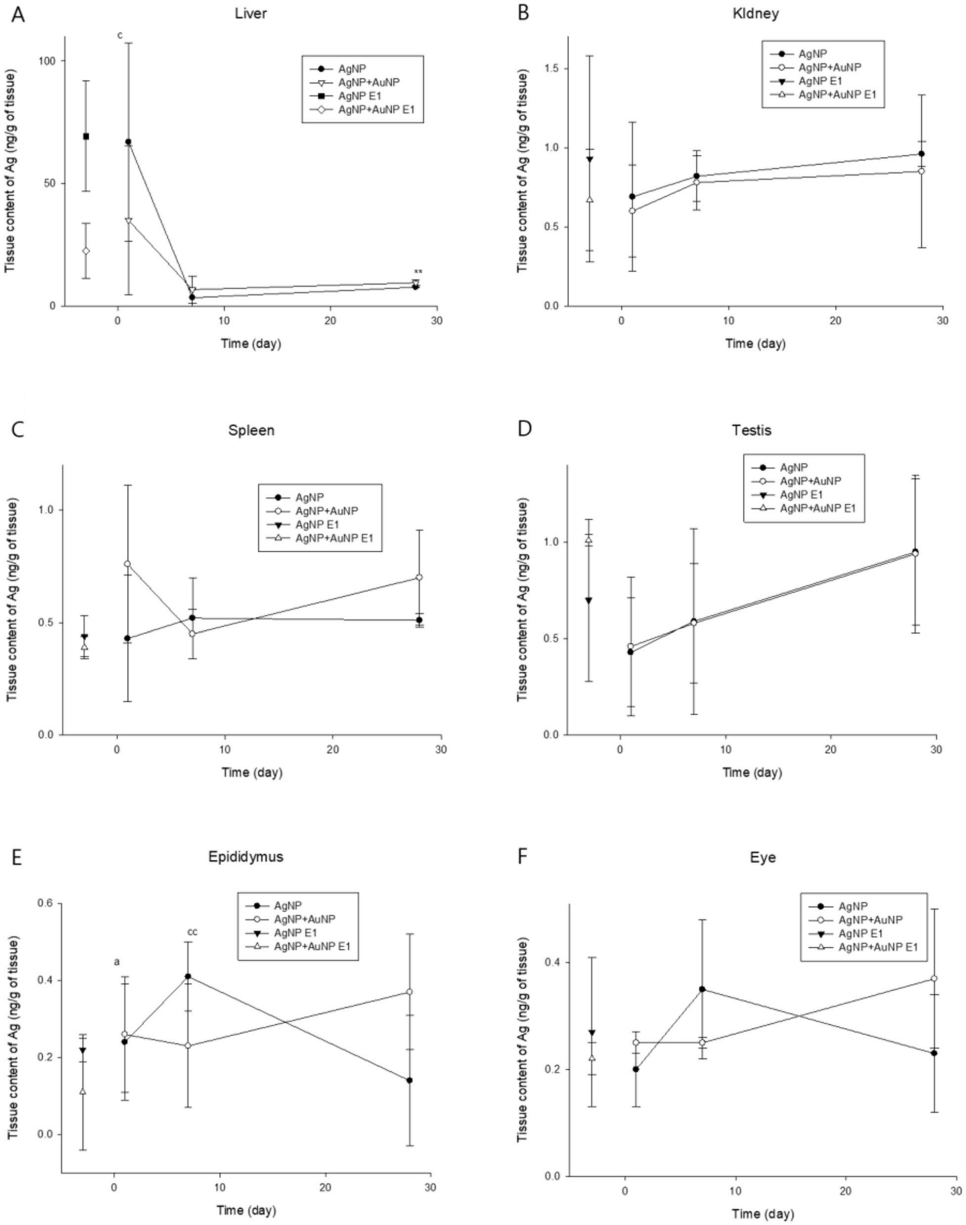

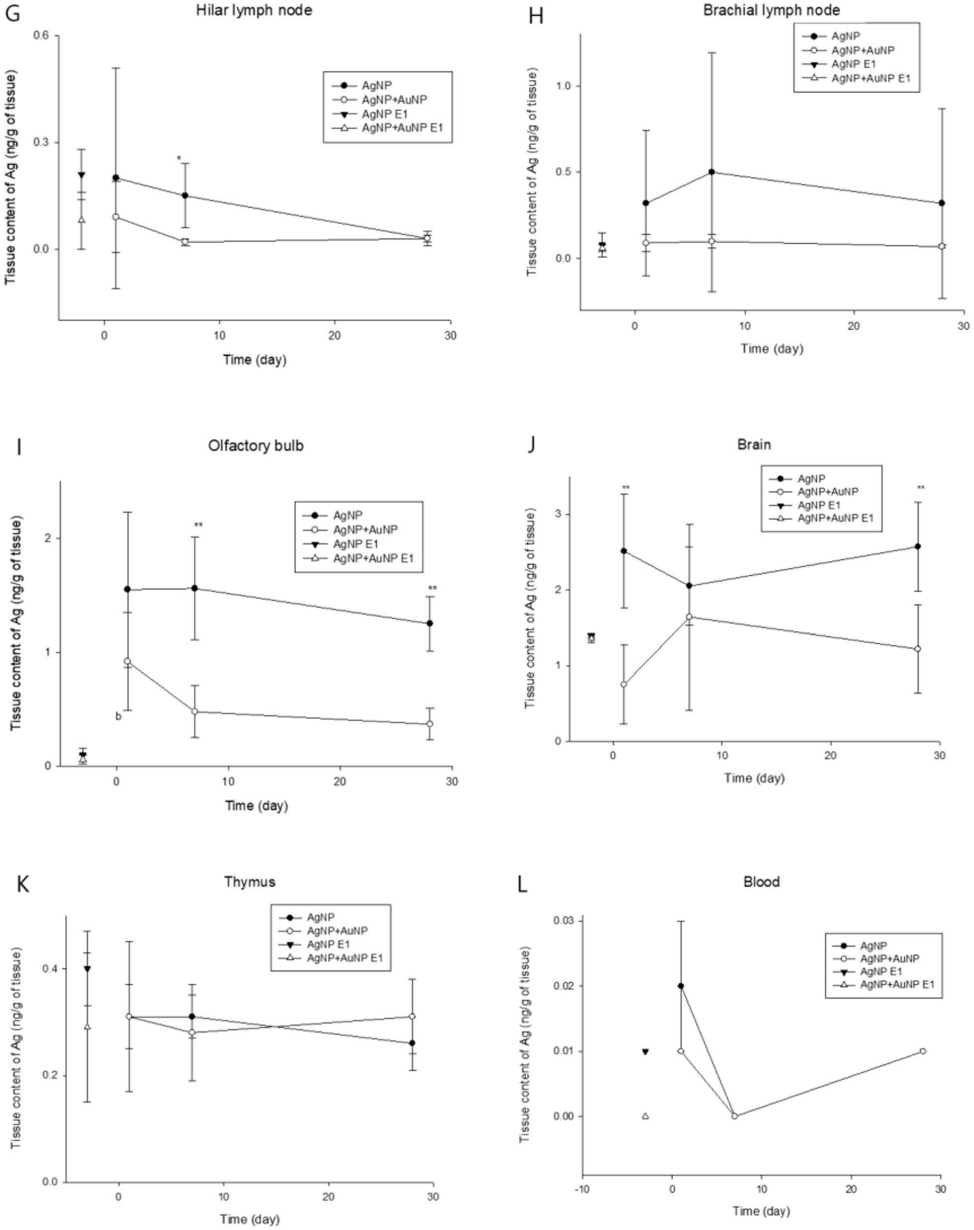
Tissue Ag concentration after 28-day inhalation exposure and post exposure period. ^a^*P* < 0.05, compared with PEO-1 and PEO-7; ^b^*P* < 0.05, compared with PEO-1 and PEO-28; ^c^*P* < 0.05, compared with PEO-7 and PEO-28; ^cc^*P* < 0.01, compared with PEO-7 and PEO-28; Where (*t*-test) *P < 0.05, and **P < 0.01

Compared with AuNP exposure, in which AuNPs were continually accumulated in the organ during 28-days of inhalation exposure and eliminated AuNP very slowly thereafter or accumulated in some tissues, AgNP exposure showed a somewhat different pattern from AuNP. The liver showed a noticeable amount of Ag that was translocated from the lung but eliminated very fast, showing T_1/2_ 1.4 days for AgNP exposure and 2.5 days for AuNP co-exposure (Table 1). The olfactory bulb and brain also showed a significant amount of translocation compared with other organs (Table 3). As shown in Fig. 2, the similar levels of Ag in the organs at E-1 comparing the levels of Ag at PEO-1 indicated that Ag in the organs was rapidly cleared from the organs. Liver (T_1/2_ 1.4 days for AgNP and 2.5 days for co-exposure) (Table 1), kidney, spleen, testis, epididymis, eye, hilar and brachial lymph nodes, and thymus showed rapid elimination of Ag from the tissue. Although the kidney, spleen, epididymis, hilar and brachial lymph nodes, and thymus showed somewhat long elimination, the levels of tissue concentration of Ag at E-1 are lower and similar to levels of PEO-1. Despite the fact that elimination half-time (T_1/2_) was estimated in these organs, the tissue concentration of these tissues was so small (< ng) to make any conclusions. Therefore, a small amount of Ag was translocated, and Ag may not be accumulated in those tissues. In contrast, the olfactory bulb and brain showed an accumulation of Ag in the organs during the post-observation period (Table 3, Additional file 1: Fig. S4). Testis and brain showed a trend of accumulation of Ag during the PEOs. The retention of Ag in most organs was not affected by the co-exposure of AuNP, except for some statistical differences in the spleen at PEO-1 and olfactory bulb at PEO-7 and 28 (Fig. 2, Table 3, Additional file 1: Fig. S4).

## Discussion

In the present work, we have studied extrapulmonary translocation and retention of Au and Ag after subacute inhalation exposure to AuNP or AgNP single exposure or AuNP + AgNP co-exposure and thereafter post-exposure observation (PEO) period. Earlier studies have shown that the inhaled AgNP was cleared from the lung by a two-phase mode, fast and slow, while AuNPs were cleared by a one-phase mode [2, 3]. The inhaled AuNP and AgNP were detected in the extrapulmonary organs were analyzed by ICP-MS. Since AuNP is insoluble, AuNP will be in the form of AuNP form. Ag translocated to the extrapulmonary organs could be either Ag ions, AgNP, or secondary AgNP, produced by silver ions reacting with biomolecules [2, 3, 5]. Whichever form AgNPs are translocated from the lung, and Ag measured in the extrapulmonary tissue were rapidly cleared from the tissues except for the olfactory bulb and brain, evidenced by similar concentration levels with under nanogram ranges between Ag levels in the E-1 and Ag levels in the PEOs. Therefore, Ag showed increased retention in the olfactory bulb and brain.

Both AuNP or AgNP can also be translocated to the extrapulmonary organs by ingestion through the gastrointestinal (GI) tract by mucociliary escalation, blood or lymphatic circulation, or through the olfactory bulb. Most organs having the reticuloendothelial system (RES), such as the liver, spleen, kidney, and lymph node, retain AuNP longer time, and they may not be eliminated fast. Furthermore, organs having a biological barrier, such as the testis and brain, also retain insoluble AuNPs or insoluble AgNP. Our subacute study indicated that the translocated AuNP might not be cleared at all in most organs.

Our previous subacute inhalation study on AuNP and AgNP single exposure and AuNP + AgNP co-exposure indicated that insoluble AuNPs were eliminated slowly from the lung, showing T_1/2_ 81.5 days, while soluble AgNP showed two modes of fast (T_1/2_ 3.1 days) and slow (T_1/2_ 48.5 days) [3]. We do not know which forms of AgNP (e.g. AgNP, Ag ion, and secondary AgNP) are translocated to extrapulmonary organs, yet a particular form of AgNP could be translocated with a low level, as seen from AuNP translocation. Our previous study on the lung retention toxicokinetic study suggested that the ionic silver form, which is eliminated rapidly from the lung, could be easily translocated to extrapulmonary organs [3]. The organs with the reticuloendothelial system, such as the liver, kidney, spleen, and lymph node, did not show long-term retention of AgNP, as seen in AuNP. Therefore, major forms of AgNP translocated to the extrapulmonary organs are silver ions which are rapidly eliminated from the organs, as seen in the liver. The most secondary AgNP generated from Ag ion reacting with biomolecules in the lung could be cleared slowly from the lung with a long half-life. On the other hand, organs having biological barriers, such as the testis and brain, may transform Ag ions to insoluble secondary AgNPs and retain insoluble AgNPs long-term. Or insoluble AgNPs may be translocated to these organs. The olfactory system also acts as a direct portal for AgNP to the brain. Previous our 12-week AgNP inhalation and 4-week and 12-week recovery study at a concentration of 49, 117, and 381 µg/m^3^ also showed rapid clearance of Ag from the liver, kidneys, and spleen and long-term retention of Ag in the brain [8].

Our other study on tissue distribution and clearance of AuNP (12.8 nm) and AgNP (10 nm) single exposure and co-exposure after subacute intravenous (IV) injection and thereafter PEO-28 recovery [15] showed similar features as well as quite different features compared with the current inhalation study. Au concentration in the tissues did not clear, as seen in Ag after 4-weeks of recovery, showing biopersistency or accumulation in the liver, kidneys, spleen, and brain after PEO-28. Co-administration of AgNP + AuNP resulted in a mutual reduction of their tissue distribution with possible competitive inhibition, and these nanoparticles could be distributed to the organs in particulate forms instead of ionic forms. These IV co-administration study results showed some similarity in the biopersistency in the tissues for AuNPs with the co-inhalation exposure study but a difference in competition in the tissue distribution, while the co-inhalation study showed independent distribution and clearance from the tissues. The absorption process in the portal of exposure may likely influence this difference. Nanoparticles are deposited in the lungs and dissolved into ions and formed into secondary nanoparticles in the case of AgNP before being distributed to extrapulmonary organs through systemic circulation or lymphatic duct. Or some AgNPs inhaled were delivered to the GI tract by mucociliary escalator absorbing to the liver by the portal vein. Rapid elimination of Ag in the liver could be due to the elimination of Ag ionic form. Moreover, AuNPs or secondary AgNPs should be able to cross the air-blood barrier and the basal lamina to reach systemic circulation. In the case of IV injection, nanoparticles can be distributed to tissues without processing and crossing these barriers. Although it is not an AuNP + AgNP co-administration study, our previous study on AgNP (10 nm) 28-day oral administration study with 100 mg/kg and 500 mg/kg body weight and thereafter 4-month recovery showed gradual clearance from the liver, kidneys, and spleen due to high dose comparing with inhalation, but biopersistency to the testis and brain [16]. Therefore, the route of administration and amount of dosing can influence tissue distribution and clearance of nanoparticles.

## Conclusions

AgNPs, (10.86 nm) and AuNPs, (10.82 nm) were exposed to male rats for 28 days (6-h/day, 5-days/week for four weeks) either with separate NP inhalation exposures or with combined co-exposure in a nose-only inhalation system. Extrapulmonary translocation from the lung and elimination from the major extrapulmonary organs were determined on day one and on post-exposure days 1, 7, and 28 (PEO-1, PEO-7, and PEO-28). AuNP was translocated to the extrapulmonary organs, including the liver, kidney, spleen, testis, epididymis, olfactory bulb, hilar and brachial lymph nodes, and brain after subacute inhalation and showed biopersistence regardless of AuNP single exposure or AuNP + AgNP co-exposure, showing similar elimination half-time. Ag was translocated to the extrapulmonary tissues and rapidly eliminated from the tissues regardless of AuNP co-exposure. Ag was continually accumulated in the olfactory bulb and brain and persistent until PEO-28. The co-exposure study of AuNP and AgNP indicated that soluble AgNP and insoluble AuNP translocated differently, showing soluble AgNP could be dissolved into Ag ion to translocate to the extrapulmonary organs and rapidly removed from most organs except the brain and olfactory bulb. Insoluble AuNPs were continually translocated to the extrapulmonary organs, and they were not eliminated rapidly.

## Materials and methods

### AuNP and AgNP aerosol generation

The method of co-inhalation exposure of AuNP and AgNP has been published [3]. The nano-aerosol generator consisted of a small ceramic heater connected to an AC power supply that was housed within a quartz tube furnace. The heater dimensions were 50 × 5 × 1.5 mm, and a surface temperature of about 1500 °C within a local heating area of 5 × 10 mm^2^ was achieved within about 10 s. For long-term testing, the source materials (about 160 mg), silver wire (100 mg, 99.99% purity, 0.5 mm diameter, Higgslab Co., Ltd, Korea), and gold wire (70 mg, 99.99% purity, 0.5 mm diameter, Higgslab Co., Ltd, Korea), were positioned in a separate ceramic heater at the highest temperature point. The quartz tube was 70 mm in diameter and 140 mm in length. Clean (dry and filtered) air was used as the carrier gas, and the gas flow was maintained at 25.0 L/min (Re = 572, laminar flow regime) using a mass flow controller (MFC, AERA, FC-7810CD-4 V, Japan) [6–8, 17]. In the current study, the exposure system consisted of four nose-only chambers; fresh air control, AgNP exposure, AuNP exposure, and AuNP + AgNP co-exposure (Additional file 1: Fig. S5). Each generator used 4–5 Lpm (liters per minute), and the remaining air flows of AgNP, AuNP, and AuNP + AgNP were 25.1 ± 0.10 Lpm, 24.8 ± 0.15, and 24.2 ± 0.1 Lpm (AgNP 11.9 ± 0.12 Lpm / AuNP 12.3 ± 0.11 Lpm), respectively. The total airflow in each chamber was 35 Lpm, controlled by the mass flow controller. The airflow from the generators was divided by a valve controller into the AgNP, AuNP, and AuNP + AgNP exposure chambers (NITC, HCT, Icheon, Korea). The target nanoparticle diameter was 10 nm for each nanoparticle exposure, and the target mass concentrations for the AgNP, AuNP, and AuNP + AgNP exposures were 20 µg/m^3^, 20 µg/m^3^, and 10 µg/m^3^ AgNP + 10 µg/m^3^ AuNP, respectively [3].

### Monitoring of inhalation chambers and analysis of AgNPs and AuNPs

In each chamber, the nanoparticle size distribution, including the count median diameter (CMD), geometric standard deviation (GSD), particle number, volume, and predicted surface area, were recorded using a differential mobility analyzer system (DMAS) comprised of a differential mobility analyzer (DMA-20, 4220, range 6–225 nm, HCT Co., Ltd. Korea) and condensation particle counter (CPC, 3775, size range 4 nm–1 μm, TSI INC., Shoreview, MN). Nanoparticles from 6 to 225 nm were measured using sheath air at 15 L/min and polydispersed aerosol air at 1.5 L/min for the DMAS with a density of 10.49 g/cm^3^ for Ag and 19.32 g/cm^3^ for Au, respectively. In addition, the mass concentrations of AgNP and AuNP were determined chemically by using an atomic absorption spectrophotometer (AAS, Perkin-Elmer 900 T, Waltham, MA, USA) after sampling on a mixed cellulose ester (MCE) filter (size: 37 mm and pore size 0.45 μm, SKC, UK) at a flow rate of 1.0 L/min and digesting the samples on a hot plate (PerkinElmer, Concord, ON, Canada) using nitric acid (Fluka, Lot; BCBM5181V). Two samples collected daily from each chamber were analyzed during the 28-day exposure period.

### Transmission electron microscopy (TEM)

The AgNPs, AuNPs, and AuNPs + AgNPs were collected on a TEM grid (electron microscope, 200 mesh, Formvar/Carbon, TEDpella, CA) and imaged for morphology using a field emission transmission electron microscope (FE-TEM, JEM2100F, 200 kV, JEOL, Tokyo, Japan). Their chemical composition was analyzed using an energy-dispersive X-ray analyzer (EDX, TM200, Oxford Instruments PLC, Oxfordshire, UK), while the CMD and GSD were obtained after measuring 200 particles for each.

### Animal care and housing conditions

Seventy-six male 6-week-old specific-pathogen-free Sprague–Dawley rats (average body weight 178.53 ± 0.63 g) were purchased from OrientBio (Seongnam, Korea) and acclimated for one week before commencing the experiments. Three to four rats were housed in polycarbonate cages during the acclimation and experimental period. The animal room temperature, humidity, and light/dark cycle were 21.40 ± 0.55 °C, 48.67 ± 5.56%, and 12 h, respectively. Filtered water and a rodent diet (BSC, Republic of Korea) were supplied ad libitum. The rats were adapted to the nose-only tubes for a week with daily tube placement for 2 h. The 7-week-old rats weighing 273.63 ± 2.83 g were divided into four groups: fresh air control, AgNP, AuNP, and AuNP + AgNP exposure groups, and exposed 6-h/day, 5 days/week for four weeks. Each exposure group included 19 animals (4 rats for day-1 (6-h) exposure and five rats for 1-day, 7-days, and 28-days post-exposure sacrifices, respectively). The animals were examined daily on weekdays for any evidence of exposure-related effects, including respiratory, dermal, behavioral, nasal, or genitourinary changes suggestive of irritation. The body weights were evaluated at the time of purchase, at the time of grouping, once a week during the inhalation exposure and post-exposure period, and before necropsy (results are not shown). The rat experiments were approved by the Hanyang University Institutional Animal Care and Use Committee in South Korea (HY-IACUC-2017-0143A).

**Fig. 3:**
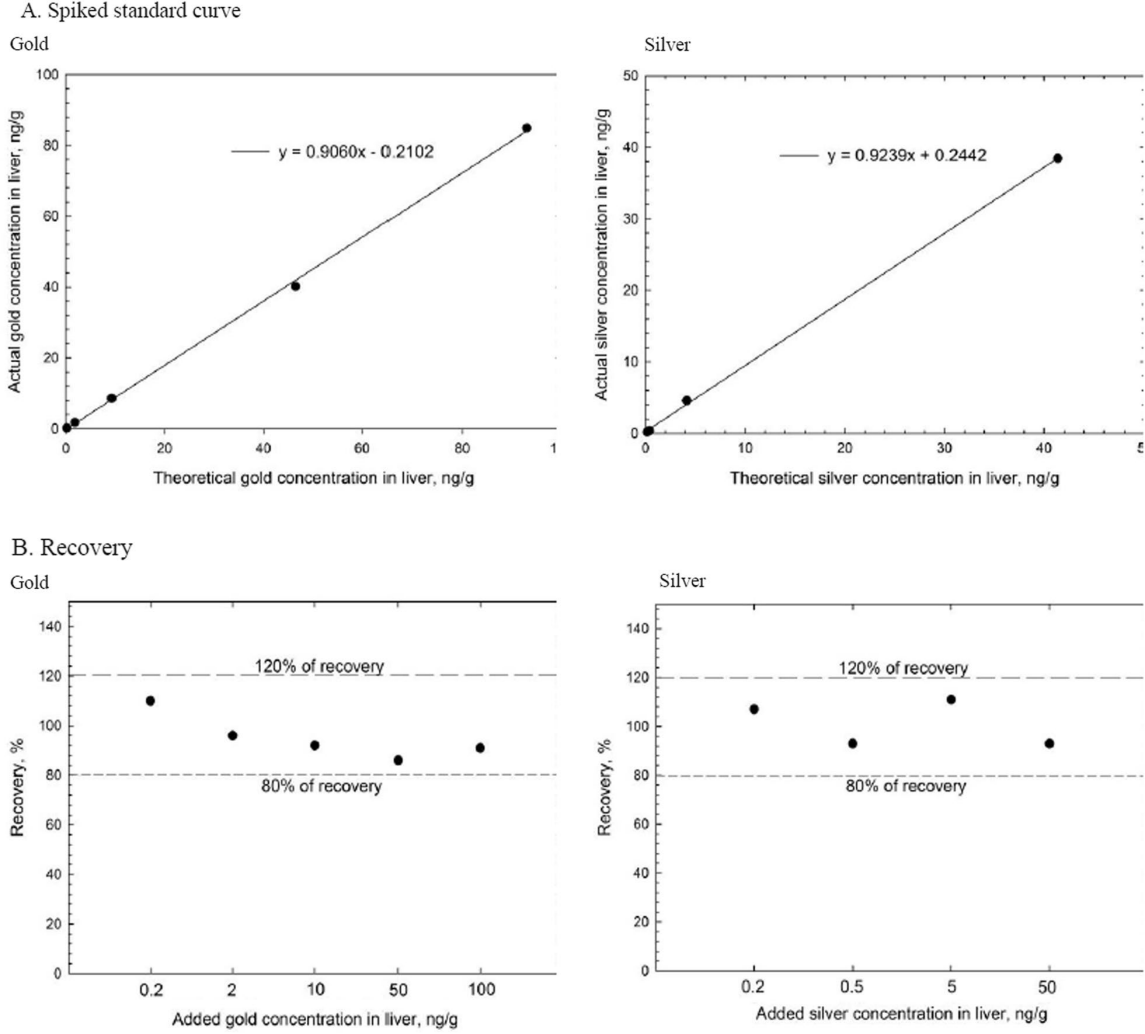
Spiked standard curve and recovery for gold and silver in liver. **A**, Spiked standard curve ranges were analyzed gold of 0.2–100 ng/g and silver of 0.2–50 ng/g. **B**, this measured gold recovery of 91–110% and silver recovery of 93–111%

Immediately after the 6-h exposure on days 1 and 1, 7, and 28 days after the 28-day exposure period, rats were sacrificed by anesthetizing via an intraperitoneal injection of pentobarbital (EntobarVR, Hanlim Pharm Co. Ltd., Seoul, Korea) at a dose of 150 mg/kg body weight. The animals in the control group were sacrificed first, and all the dissection instruments were thoroughly washed with 70% ethyl alcohol in between the dissections to avoid NP contamination from one organ to another. Blood was drawn from the abdominal aorta for exsanguination. Lungs, liver, kidneys, spleen, testis, epididymis, thymus, hilar lymph node, bronchial lymph node, olfactory bulb, brain, and eyes were selected. After measuring the organ weights, the organs were fixed with 10% neutral buffer formalin for further processing. An aliquot of the fixed organs was then digested as described in NIOSH 7302 [18] using a microwave (MARS 230/60, CEM, Matthews, NC) with the following three steps: (1) increase the temperature to 110 °C for 15 min; (2) maintain this temperature for 60 min (1600 w); and (3) cool for 15 min. The digestion solution for lung tissue consisted of 2 mL of nitric acid (purity of 69.0%; CAS. No of 7697–37-2, Fluka, Germany), and 3 mL of 1% nitric acid to make a final volume of 5 ml. The samples were then analyzed using an inductively coupled plasma mass spectrometer (ICP-MS, PerkinElmer NEXION 300S, Concord, ON, Canada). The ICP-MS analysis was conducted according to NIOSH 8200 [19]. The concentrations of Ag and Au in the organs were determined by ICP-MS based on standard curves established with un-exposed clean livers spiked with test NPs sampled from the respective inhalation chambers, where the results from digestion, extraction, and dilution were all performed in duplicates. The quantitative analyses for Ag and Au in the liver were corrected using the spiked standard curve. The recovery yields of AgNPs and AuNPs were 81 – 113% and 84 – 105%, respectively, as shown in Fig. 3. The spiked standard curves ranged from 0.2 – 5 ng/g of liver tissue for AgNPs and 2–100 ng/g of lung tissue for AuNPs. When analyzing the samples, the dilution factor was 100 times. The digestion recovery of AgNPs and AuNPs in the liver tissue was calculated using Eq. 1

1 $${\text{Recovery }}\;\left( {\text{\% }} \right) = {\text{measured}}\;{\text{ concentration}}\; {\text{(ng/g)/spiked}}\;{\text{concentration }}\;{\text{(ng/g)}} \times {1}00$$

The mass content of nanoparticles in the organ was calibrated using the weight of the organ. The samples were all analyzed using a standard calibration curve that ranged from 0.05 – 0.5 ppb for Ag and 1–10 ppb for Au. After analyzing standard blanks 40 times, the measured LOD and LOQ were 0.086 µg /L and 0.260 µg /L, respectively, for Ag and 0.027 µg /L and 0.082 µg/L, respectively, for Au.

### Organ retention, translocation, and elimination kinetics

The organ elimination kinetics for the AgNPs, AuNPs, and AuNP + AgNP co-exposure were determined based on lung burdens measured on 1-day (6-h) of exposure (E-1) and on post-exposure observation days 1 (PEO-1), 7 (PEO-7), and 28 (PEO-28). The fraction of organ concentration per initial organ concentration at PEO-1 was used for estimating retention, translocation, and clearance kinetics for PEO-7 and PEO-28, applying an appropriate-order clearance model. The fractions of organ concentration at PEO-1 (i.e. PEO-1/PEO-1, PEO-7/PEO-1 and PEO-28/PEO-1) were plotted as Y-axis, and PEO periods were plotted as the X-axis. The -order model is described by Eq. 2. The retention half-time () was derived using 1, 2, and natural log (2), as shown in Eq. (3).

2 $${\text{M}}\left( {\text{t}} \right) = {\text{P }}exp\left( { - \lambda t} \right)$$

where

- M(t); lung burden at time (t)
- P; fraction of lung burden cleared (1.0 for one-compartment model)
- $\lambda$; clearance rate per day for one-compartment model
  3 $${\text{T}}_{{1/2{ }}} = { }\frac{{{\text{ln}}\left( 2 \right)}}{\lambda } \approx \frac{0.693}{\lambda }$$

### Statistical analysis

An analysis of variance (ANOVA) test and Dunnett T3 multi-range tests were used with up to two points, where one point compared the single and co-exposure groups, while two points compared each group from PEO-1 to PEO-28. The level of significance was set at *P* < 0.05.

## Supplementary Information

**Additional file 1**. **Table S1**: Aerosol data for AuNPs, AgNPs, and AuNP + AgNP co-exposure (From Table 1 of Kim et al. [3]). **Figure S1**: FE-TEM analysis for AgNPs, AuNPs, and AgNP + AuNP co-exposure in chambers [3; A, image of single AgNP (scale 20 nm); B, EDS result for single AgNP; C, image of single AuNP (scale 20 nm); D, EDS result for single AuNP; E, image of AgNP + AuNP co-exposure (scale 100 nm); F, EDS result for AgNP + AuNP co-exposure. **Figure S2**: Particle distribution in exposure chambers based on DMAS and FE-TEM [3]; (A), CMD and GSD using DMAS during exposure period; (B), Particle diameter using DMAS; (C), CMD and GSD for AgNPs using FE-TEM; (D), CMD and GSD for AuNPs using FE-TEM; (E) CMD and GSD for AgNP + AuNP co-exposure using FE-TEM. **Table S2**: Lung burden of AuNPs, AgNPs and AuNsP + AgNPs co-exposure (ng/lung). **Figure S3**: Au retention after 28-day inhalation exposure and post-exposure period. **Figure S4**: Ag retention after 28-day inhalation exposure and post-exposure period. **Figure S5**: Schematic of exposure system for generating AuNPs, AgNPs, and AuNP + AgNP co-exposure for nose only exposure chambers [3]

## Data Availability

All data and materials are included in the manuscript, tables, figures and supplements. The datasets during and/or analyzed during the current study are available from the corresponding author on reasonable request.
