# Supplementary material for: Biokinetics of subacutely co-inhaled same size gold and silver nanoparticles
Source: Part Fibre Toxicol. 2023 Mar 31;20:9. doi: 10.1186/s12989-023-00515-z (PMC10064767; doi:10.1186/s12989-023-00515-z)

**[Supplement]**

Fate of gold and silver nanoparticles in organs after subacute co-inhalation exposure of gold and silver nanoparticles with similar sizes

^1^Philku Lee, ^1^Jin Kwon Kim, ^1^Mi Seong Jo, ^1^Hoi Pin Kim, ^2^Kang Ho Ahn, ^3^Jung Duck Park ^4,5^Mary Gulumian, ^6^Günter Oberdörster* and ^1^Il Je Yu*

^1^HCT CO.,LTD. Icheon, Korea ^2^Department of Mechanical Engineering, Hanyang University, Ansan, Korea; ^3^College of Medicine, Chung-Ang University, Seoul, Korea, ^4^Haematology and Molecular Medicine, University of the Witwatersrand, Johannesburg, South Africa, ^5^Water Research Group, Unit for Environmental Sciences and Management, North-West University, Potchefstroom, South Africa ^6^Department of Environmental Medicine, University of Rochester, Rochester, NY, USA

*Corresponding authors: Il Je Yu, HCT CO.,LTD. Seoicheon-ro 578 beon-gil, Majang-myeon, Icheon, 17383, Korea, Email: [u1670916@chollian.net](mailto:u1670916@chollian.net),  Tel: 031.645.6358, Fax: 031.645.6358

Supplement 1. Aerosol data for AuNPs, AgNPs, and AuNP+AgNP co-exposure (From Table 1 of Kim et al [3])


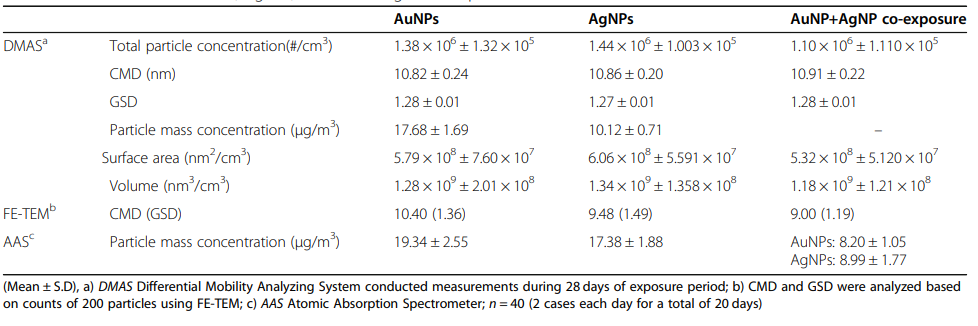


Supplement 2. FE-TEM analysis for AgNPs, AuNPs, and AgNP+AuNP co-exposure in chambers (Kim et al., 2021); A, image of single AgNP (scale 20 nm); B, EDS result for single AgNP; C, image of single AuNP (scale 20 nm); D, EDS result for single AuNP; E, image of AgNP+AuNP co-exposure (scale 100 nm); F, EDS result for AgNP+AuNP co-exposure.


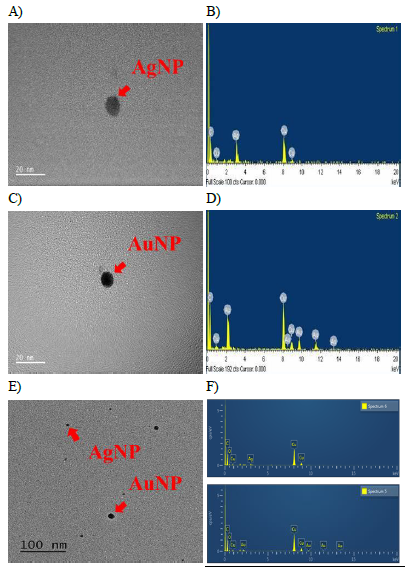


Supplement 3. Particle distribution in exposure chambers based on DMAS and FE-TEM (Kim et al., 2021); (A), CMD and GSD using DMAS during exposure period; (B), Particle diameter using DMAS; (C), CMD and GSD for AgNPs using FE-TEM; (D), CMD and GSD for AuNPs using FE-TEM; (E) CMD and GSD for AgNP+AuNP co-exposure using FE-TEM.


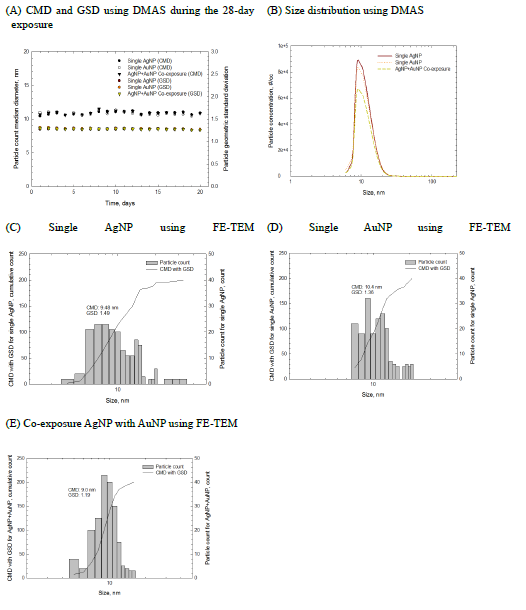


Supplement 4. Lung burden of AuNPs, AgNPs and AuNsP+AgNPs co-exposure (ng/lung)


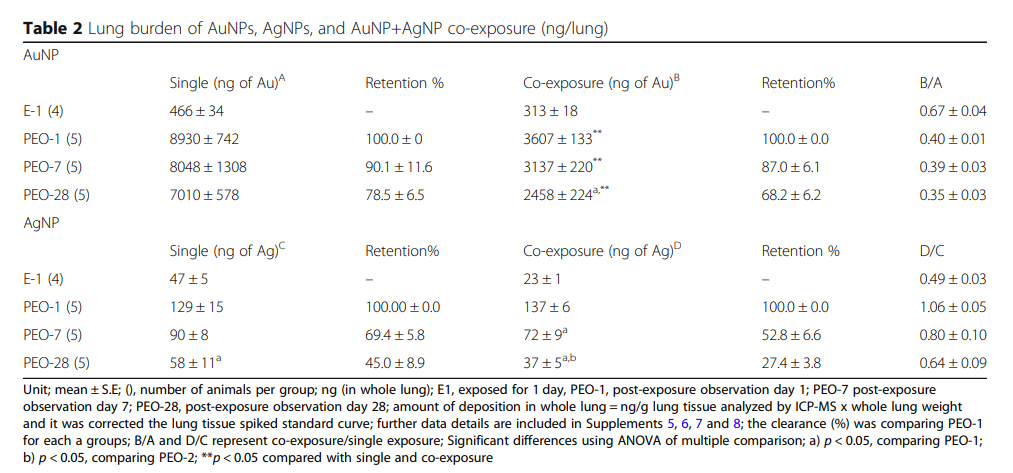


Supplement 5. Au retention after 28-day inhalation exposure and post-exposure period. The data were plotted as tissue concentrations of PEO-1/PEO-1, PEO-7/PEO-1, and PEO-28/PEO-1.

| A | B |
| --- | --- |
|  |  |
| C | D |
|  |  |
| E | F |
|  |  |
| G | H |
|  |  |
| I | J |
|  |  |
| K | L |
|  |  |

* P<0.05, and ** P<0.01 comparing AuNP with AuNP+AgNP

Supplement 6. Ag retention after 28-day inhalation exposure and post-exposure period. The data were plotted as tissue concentrations of PEO-1/PEO-1, PEO-7/PEO-1, and PEO-28/PEO-1.

| A | | B | |
| --- | --- | --- | --- |
|  | |  | |
| C | | D | |
|  | |  | |
| E | | F | |
|  | |  | |
| G | H | |  |
|  |  | |  |
| I | J | |  |
|  |  | |  |
| K | L | |  |
|  |  | |  |

* P<0.05, and ** P<0.01 comparing AgNP with AuNP+AgNP

Supplement 7. Schematic of exposure system for generating AuNPs, AgNPs, and AuNP+AgNP co-exposure for nose only exposure chambers (Kim et al., 2021)


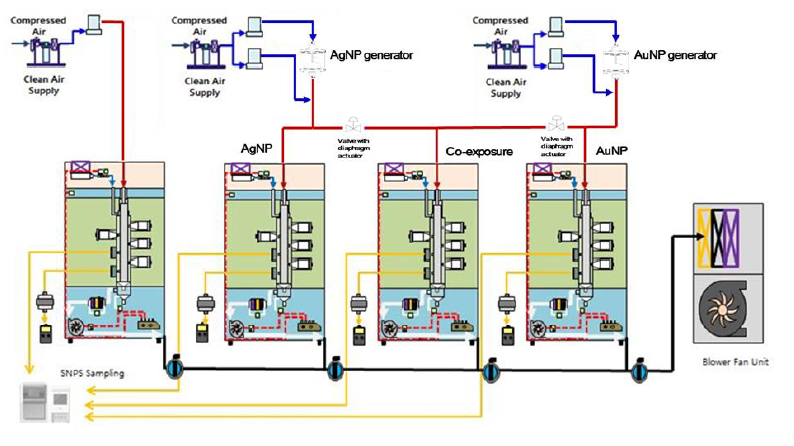

Supplement: Supplementary file 1 — Additional file 1. Table S1: Aerosol data for AuNPs, AgNPs, and AuNP + AgNP co-exposure (From Table 1 of Kim et al. [3]). Figure S1: FE-TEM analysis for AgNPs, AuNPs, and AgNP + AuNP co-exposure in chambers [3; A, image of single AgNP (scale 20 nm); B, EDS result for single AgNP; C, image of single AuNP (scale 20 nm); D, EDS result for single AuNP; E, image of AgNP + AuNP co-exposure (scale 100 nm); F, EDS result for AgNP + AuNP co-exposure. Figure S2: Particle distribution in exposure chambers based on DMAS and FE-TEM [3]; (A), CMD and GSD using DMAS during exposure period; (B), Particle diameter using DMAS; (C), CMD and GSD for AgNPs using FE-TEM; (D), CMD and GSD for AuNPs using FE-TEM; (E) CMD and GSD for AgNP + AuNP co-exposure using FE-TEM. Table S2: Lung burden of AuNPs, AgNPs and AuNsP + AgNPs co-exposure (ng/lung). Figure S3: Au retention after 28-day inhalation exposure and post-exposure period. Figure S4: Ag retention after 28-day inhalation exposure and post-exposure period. Figure S5: Schematic of exposure system for generating AuNPs, AgNPs, and AuNP + AgNP co-exposure for nose only exposure chambers [3] [file 12989_2023_515_MOESM1_ESM.docx]
